# Targeted degradation of CBP/p300 by CBPD-409 exhibits robust anti-myeloma activity

**DOI:** 10.1038/s41375-026-03004-2

**Published:** 2026-06-16

**Authors:** S. Corradini, M. Maksimos, F. Azab, S. Arsalani, E. Nwarunma, G. Watt, R. Soutar, M. Powell, R. Henderson, J. Travers, Z. Chen, S. Wang, A. K. Azab, M. T. S. Williams

**Affiliations:** 1 Research Centre for Health, School of Health and Life Sciences, Department of Biological and Biomedical Sciences, Glasgow Caledonian University, Glasgow, UK; 2 Department of Biomedical Engineering, University of Texas Southwestern, Dallas, TX USA; 3 The Beatson West of Scotland Cancer Centre, Glasgow, UK; 4 Department of Hematology, Fujian Institute of Hematology, Fujian Provincial Key Laboratory on Hematology, Fujian Medical University Union Hospital, Fujian, China; 5 Department of Internal Medicine, Division of Hematology & Oncology, University of Michigan Medical School, Ann Arbor, MI, USA Rogel Cancer Center, The University of Michigan - Michigan Medicine, Ann Arbor, MI, USA Department of Pharmacology, Department of Medicinal Chemistry, University of Michigan Medical School, Ann Arbor, MI USA; 6 School of Cancer Sciences, College of Medical, Veterinary and Life Sciences, University of Glasgow, Glasgow, UK

**Keywords:** Targeted therapies, Myeloma

Epigenetic regulation is central to multiple myeloma (MM) pathogenesis. Among the most critical regulators are the histone acetyltransferases CREB‑binding protein (CBP) and p300, which act as transcriptional co‑activators that maintain MYC‑driven proliferation, IRF4‑dependent survival, and plasma cell identity [1]. Pharmacologic inhibition of CBP/p300 bromodomains with CCS1477/inobrodib has shown early clinical activity in a phase 2 trials (NCT04068597) for MM, confirming these co‑activators as actionable targets [2]. However, inhibitors act via occupancy‑driven pharmacology, suppressing only catalytic function while leaving intact the non‑enzymatic scaffolding roles that are critical for oncogenic transcription [3]. As a result, their efficacy is incomplete.

Proteolysis‑targeting chimeras (PROTACs) offer a fundamentally distinct approach by exploiting the ubiquitin-proteasome system to eliminate proteins altogether. PROTACs are bifunctional molecules that simultaneously bind the protein of interest and recruit an E3 ubiquitin ligase, leading to ubiquitination and proteasomal degradation [4]. This “event‑driven” modality results in complete and sustained target loss, abolishing both enzymatic activity and non‑canonical scaffolding functions. Our previous studies demonstrate that PROTACs have therapeutic potential in MM [5], with other researchers showing the promise of PROTACs in Leukemia [6]. Importantly, a recent study demonstrated that CBP/p300 degraders impose distinct transcriptional consequences compared with inhibitors, producing more profound and functionally biased repression of oncogenic gene programs [3]. CBPD‑409 is a potent and selective CBP/p300 PROTAC that is composed of GNE-049, a bromodomain inhibitor, and thalidomide a CRBN ligand (Fig. 1A).

**Fig. 1:**
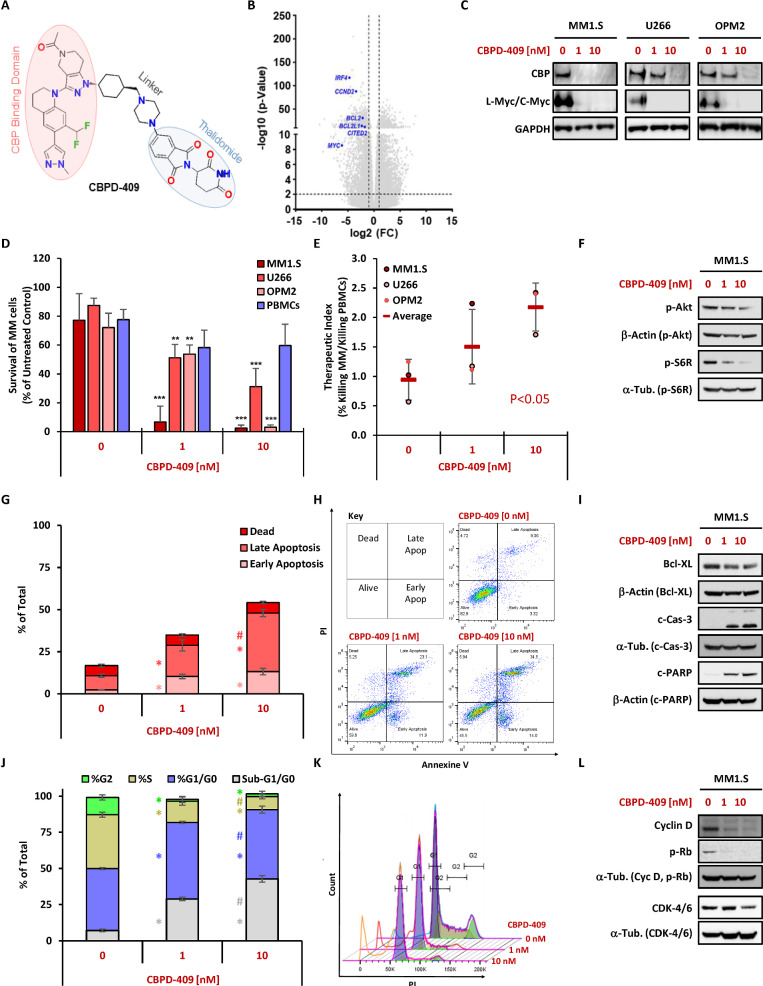
CBPD-409 exhibits cytotoxic and cytostatic effects in MM cells via collapse of pro-survival and proliferative pathways*.* CBPD-409 chemical structure with the bromodomain-binding warhead, linker, and cereblon-recruiting thalidomide moiety highlighted **A**. Volcano plot of RNA-seq from MM1.S cells treated with CBPD-409 for 24 h with major myeloma drivers annotated, based on 6 independent experiments (*n* = *6*), statistical tests were two tailed t-tests with the assumption of equal variances **B**. Immunoblot of CBP, L-MYC, and C-MYC in MM1.S, U266, and OPM-2 after treatment with CBPD-409 (1-10 nM) or DMSO for 24 h, GAPDH loading control **C**. Survival of MM1.S, U266, OPM-2, and PBMCs after 72 h exposure to CBPD-409 (1-10 nM) or matched DMSO quantified by Annexin V and fixable viability dye flow cytometry **D**. Therapeutic index calculated as myeloma cell killing divided by PBMC killing following treatment with CBPD-409 (0-10 nM). Statistical analysis was performed using one-way ANOVA; ** *p* < 0.01 and *** *p* < 0.001 compared to 0 nM (DMSO) **E**. Immunoblot of p-AKT and pRS6 in MM1.S after 24 h treatment **F**. Distribution of early apoptosis, late apoptosis, and dead fractions in MM1.S after 72 h CBPD-409 (1-10 nM) or DMSO assessed by Annexin V/FVD (*n* = 3) **G**. Statistical analysis was performed using two-tailed t-tests assuming equal variance; * compared to 0 nM *p* < 0.01, # compared to 1 nM p < 0.01. Representative gating strategy is shown **H**. Immunoblot of BCL-xL, c-caspase-3, and c-PARP in MM1.S after 24 h treatment **I**. Quantification of cell cycle phase distr**i**bution of MM1.S after 72 h under the same conditions assessed by PI staining (*n* = 3) **J**. Statistical analysis was performed using two-tailed t-tests assuming equal variance; * compared to 0 nM *p* < 0.01, # compared to 1 nM *p* < 0.01 with representative cell cycle histograms plot analysis (overlay) shown **K**. Immunoblot of Cyclin D, pRb, and CDK4/6 in MM1.S after 24 h with α- or β-tubulin loading controls **I**. Bar graphs: SD± (*n* = 3) for each cell line. AnV Annexin V, BCL-xL BCL2-like 1, c-CASP3 cleaved caspase-3, c-PARP cleaved poly(ADP-ribose) polymerase, CBP CREB-binding protein, CDK4/6 cyclin-dependent kinase 4/6, DMSO dimethyl sulfoxide, GAPDH glyceraldehyde-3-phosphate dehydrogenase, PBMCs peripheral blood mononuclear cells, PI propidium iodide, p-AKT phosphorylated AKT, p-Rb phosphorylated retinoblastoma protein, p-S6 phosphorylated ribosomal protein S6, Rb retinoblastoma protein.

Given the central role of CBP/p300 in maintaining MYC and IRF4‑driven dependencies, we hypothesized that CBPD‑409 would achieve excellent transcriptional repression and therapeutic efficacy in MM.

Here we provide preclinical evidence that CBPD‑409 exerts robust anti‑myeloma activity across in vitro and in vivo models of myeloma, highlighting degradation of CBP/p300 as an attractive therapeutic strategy in MM.

Human MM cell lines U266, MM1.S and OPM-2, together with healthy donor peripheral blood mononuclear cells (PBMCs), were exposed to CBPD-409 for 72 h across 1-10 nM to define cytostatic and cytotoxic activity and to estimate the therapeutic index. Protein levels of CBP, c-MYC in MM1.S and OPM-2, and L-MYC in U266 were examined by immunoblot after 24 h.

Mechanistic effects in MM1.S were profiled by probing p-AKT, p-S6, BCL-XL, cleaved caspase-3, cleaved PARP, Cyclin D, p-Rb and CDK4/6. Viability and apoptosis were quantified by Annexin V with Fixable Viability Dye, and cell cycle analysis and proliferation by propidium iodide flow cytometry. Anti-tumor activity in vivo was tested in MM1.S-CBR-GFP xenografts treated with daily oral CBPD-409 at 3 mg/kg or vehicle for 21 days, with tumor burden monitored by serial bioluminescence imaging. Global transcriptomic analysis was assessed by RNA-seq in MM1.S after 24 h of exposure to CBPD-409 or its matched vehicle control (DMSO).

RNA-seq revealed co-ordinated suppression of core myeloma oncogene programmes, including *MYC, IRF4, CCND2, CITED2, BCL2 and BCL2L1 (BCL-xL)*, consistent with the role of CBP and p300 in sustaining enhancer driven IRF4-MYC circuitry in myeloma [7] (Fig. 1B).

CBPD-409 produced rapid and near complete loss of CBP in MM cells. At 10 nM, CBP protein expression fell by a mean of 95% ± 5 SEM at 24 h in U266, MM1.S and OPM-2, with concordant depletion of lineage MYC paralogs, L-MYC in U266 and c-MYC in MM1.S and OPM-2 (Fig. 1C).

Survival declined in a concentration-dependent manner across 1-10 nM, importantly with minimal effects in healthy PBMCs at matched concentrations, yielding an increasingly favourable therapeutic index even at higher concentrations (Fig. 1D, E). MM1.S showed the greatest sensitivity and was therefore selected for deeper mechanistic profiling. In MM1.S cells, CBPD-409 reduced phosphorylation of AKT and S6, indicating suppression of pro-survival PI3K/AKT/mTOR signaling downstream of treatment (Fig. 1F). Apoptosis was prominent at 10 nM, with increased early and late apoptotic fractions and a corresponding expansion of Annexin V-positive populations by flow cytometry (Fig. 1G, H). Immunoblotting corroborated this apoptotic phenotype, showing reduced BCL-XL together with increased cleaved caspase-3 and cleaved PARP (Fig. 1I). Cell-cycle analysis further showed depletion of S and G2/M fractions, accompanied by increased G1/G0 and sub-G1/G0 populations (Fig. 1J). This shift was also evident in the propidium iodide signal (Fig. 1K). Consistent with G1-associated cell-cycle suppression, CBPD-409 reduced cyclin D, p-Rb and CDK4/6 levels in MM1.S cells (Fig. 1L), quantified in Supplementary Fig. 1.

Together, these findings provide mechanistic insight into CBPD-409-driven pro-apoptotic signaling and G1-associated cell-cycle arrest in MM cells.

In vivo, CBPD-409 induced marked tumour regression in MM1.S-CBR-GFP xenografts, with seven-fold, 54-fold and 908-fold reductions in bioluminescence on days 7, 14 and 21 versus vehicle (Fig. 2A, B).

**Fig. 2:**
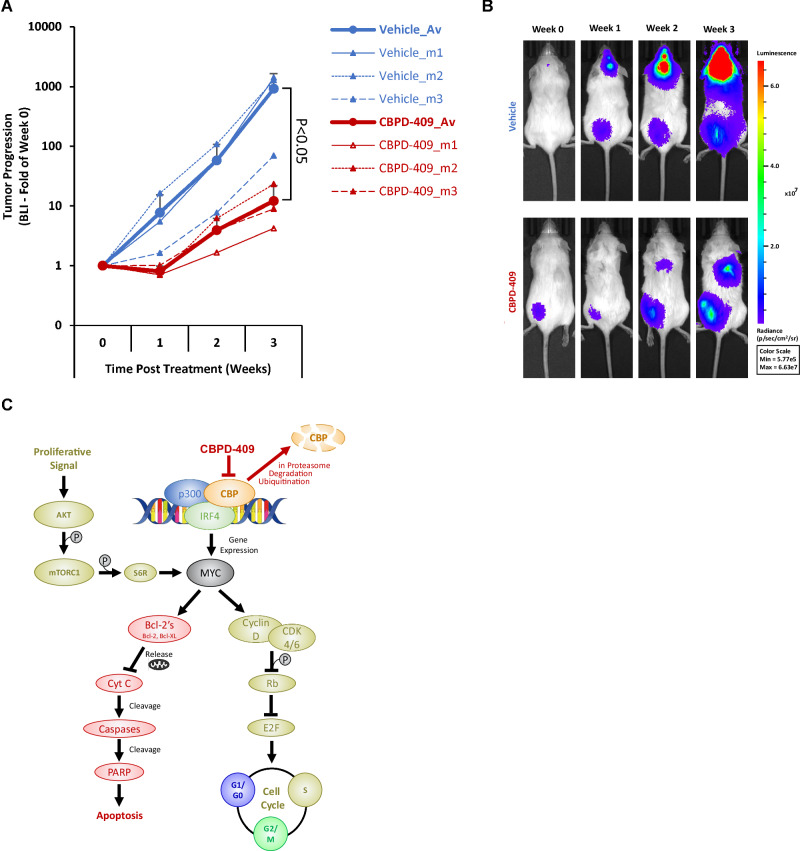
CBPD-409 suppresses myeloma tumour growth In vivo. MM1.S xenograft mice were treated with vehicle (100% PEG2000) or 3 mg/kg of CBPD-409 daily for 21 days (*n* = 3 per group), statistical analysis was performed using two-way ANOVA; *p* < 0.05 **A**. MM disease progression was assessed by Bioluminescent Imaging (BLI), m = mouse, Av = average BLI value/MM burden. BLI - Fold of week 0 = Fold change in luminescent signal relative to baseline measurements taken at treatment initiation. **B** Representative BLI images. **C** CBPD-409 recruits Cereblon (E3 ligase) to CBP/p300 to form a ternary complex that triggers ubiquitination and proteasomal degradation of both co-activators. Loss of CBP/p300 reduces H3K27ac at super-enhancers, dampens IRF4-MYC transcriptional output, and curtails downstream proliferative signaling. The schematic shows repression of the Cyclin D-CDK4/6-pRb-E2F axis leading to G1 arrest, together with reduced expression of pro-survival BCL-2 family members that favors mitochondrial cytochrome-c Cyt C release, caspase activation, and PARP cleavage. Degradation therefore collapses enhancer-dependent survival and growth programmes and is expected to blunt AKT-mTORC1-RPS6 activity that sustains biomass and cell-cycle entry [3]. CBP CREB-binding protein (CREBBP, lysine acetyltransferase), p300 histone acetyltransferase EP300, IRF4 interferon regulatory factor 4, MYC proto-oncogene transcription factor MYC, AKT protein kinase B, mTORC1 mechanistic target of rapamycin complex 1, S6/RPS6 ribosomal protein S6, BCL-2 B-cell lymphoma 2, BCL-xL BCL-2 (BCL2L1), Cyclin D D-type cyclins (CCND1/2/3), CDK4/6 cyclin-dependent kinases 4 and 6, Rb/pRb retinoblastoma tumour-suppressor protein, E2F E2F family transcription factors, Cyt C cytochrome-c, PARP poly(ADP-ribose) polymerase.

The mechanism schematic summarises an event-driven collapse of the CBP-p300-IRF4 enhancer complex that licenses the IRF4-MYC feed-forward circuit in MM (Fig. 2C). In MM1.S cells, rapid CBP loss is followed by transcript-level repression of *MYC, CCND2, CITED2, BCL2 and BCL2L1* and by reduced MYC protein, which fits a model in which IRF4-occupied super-enhancers require CBP and p300 acetyltransferase activity to maintain chromatin accessibility and BRD4-dependent elongation. Disassembly of this platform explains the protein changes we observed, including reduced p-AKT and p-S6, lower Cyclin D and CDK4/6, pRb and diminished E2F output, yielding G1 accumulation, with apoptosis evidenced by reduced expression of BCL-XL and cleavage of caspase-3 and PARP.

Two main mechanistic considerations can be observed here. First, targeting the enhancer hub upstream of IRF4 and MYC should generate positive feedback collapse rather than partial attenuation, predicting stronger pathway convergence than potential catalytic inhibition. This suggests pharmacodynamic biomarkers, such as early loss of H3K27ac at IRF4 and MYC loci with concordant MYC and CCND2 downregulation [7].

Second, because IRF4-MYC programmes couple nutrient sensing to mTORC1 and ribosomal biogenesis, enhancer decommissioning offers a transcriptional route to dampen AKT-mTOR signaling without direct kinase inhibition, a feature that may favor combinations that spare T-cell fitness while closing escape through proliferative rebound [8]. These inferences are consistent with work defining IRF4 addiction in MM, the super-enhancer control of MYC in MM1.S, and recent evidence that interfering with p300 activity cooperates with IMiDs to depress IRF4 and MYC, and widen the therapeutic window [7].

Collectively, the data argue for CBPD-409 as the preferred way to target and abolish CBP and p300 function in enhancer-addicted myeloma. Degradation removes catalytic and scaffold activities, collapses MYC- and IRF4-linked oncogenic programmes, and, unlike bromodomain inhibition, ablates enhancer acetylation marks that sustain lineage identity. This pattern mirrors results in other CBP/ p300-driven cancers where dual CBP and p300 degradation suppressed oncogenic transcription and is well tolerated with no significant signs of toxicity, as evidenced from extensive toxicology animal studies (immune-competent CD-1 mice and rats) with CBPD-409 [3, 9]. Murine cereblon (CRBN) is known to bind immunomodulatory drugs (IMiDs) inefficiently due to species-specific amino acid differences, which limits the ability of standard mouse models to fully recapitulate CRBN-dependent pharmacology [10]. Accordingly, the xenograft studies presented here, performed in immunodeficient mice, primarily assess the tumor-intrinsic efficacy of CBPD-409 rather than systemic or host-mediated toxicity. Importantly, the tolerability of CBPD-409 has been evaluated in a humanized CRBN context.

Luo et al. demonstrated effective CBP/p300 degradation in humanized Crbn^V380E/I391V^ mice [11] without evidence of overt toxicity, including absence of weight loss, organ pathology, or clinical adverse effects [3]. These findings support the tolerability of systemic p300 degradation in a human CRBN-expressing setting. Oral exposure enables short pulses that drive deep target loss and rapid pathway convergence. Such scheduling can be paired with pharmacodynamic readouts, including loss of enhancer acetylation and early MYC suppression, to individualize dose and duration [12].

As CBPD-409 recruits cereblon via a thalidomide handle (Fig. 1A), the ligase becomes a point of leverage and of risk. IMiDs may cooperate through IKZF1 and IKZF3 co-depletion yet can also compete for CRBN, so sequencing and spacing should be guided by direct measures of degradation, and by neosubstrate surveillance that has become standard for CRBN-based degraders [13]. Proteasome integrity is required for event-driven removal of CBP and p300. Concurrent bortezomib attenuates PROTAC activity despite efficient ubiquitination, so avoidance or strict temporal separation with biochemical confirmation of proteasome recovery is advised [14]. Comparison with pharmacologic CBP/p300 inhibition will be an important priority for future work. Although inobrodib/CCS1477, which has shown promising single-agent activity in multiple myeloma, targets bromodomain function, CBPD-409 removes the full CBP/p300 protein complex and may therefore suppress non-catalytic and scaffolding functions in addition to enzymatic activity [2]. Direct comparison of these modalities in matched in vitro and in vivo settings will be necessary to determine whether degradation confers more durable repression of IRF4-driven transcriptional programmes and superior therapeutic benefit.

The apparent sparing of PBMCs should also be interpreted with caution, as viability was assessed in unstimulated PBMC cultures, where limited proliferation may reduce sensitivity for detecting cytotoxic effects. In this context, the PBMC data are best viewed as an initial indication of relative selectivity rather than a definitive assessment of toxicity, which will require orthogonal evaluation in future studies.

All together these novel findings, along with previous results, show that CBPD-409 exhibits features highly favourable for clinical use. These include its high potency, efficacy at very low doses, high selectivity with a lower chance of on-target/off-tumour side effects, its oral bioavailability, and acceptable toxicity profile. Consequently, CBPD-409 demonstrates real potential to be translated into a clinical treatment for myeloma patients.

## Supplementary information

Supplementary Fig.1 Western blot quantification of CBPD-409 on oncogenic signaling, proliferation, cell cycle progression, and apoptosis pathways in multiple myeloma cells.

## References

[1] Zhu Y, Wang Z, Li Y, Peng H, Liu J, Zhang J, et al. The Role of CREBBP/EP300 and Its Therapeutic Implications in Hematological Malignancies. Cancers. 2023;15:1219. 10.3390/cancers15041219 PMC9953837 36831561

[2] Nicosia L, Spencer GJ, Brooks N, Amaral FMR, Basma NJ, Chadwick JA, et al. Therapeutic targeting of EP300/CBP by bromodomain inhibition in hematologic malignancies. Cancer Cell. 2023;41:2136–53.e13. 37995682 10.1016/j.ccell.2023.11.001

[3] Luo J, Chen Z, Qiao Y, Tien JC, Young E, Mannan R, et al. Targeting histone H2B acetylated enhanceosomes via p300/CBP degradation in prostate cancer. Nat Genet. 2025;57:2468–81. 41044247 10.1038/s41588-025-02336-6 PMC12513837

[4] Cordani N, Nova D, Sala L, Abbate MI, Colonese F, Cortinovis DL, et al. Proteolysis Targeting Chimera Agents (PROTACs): New Hope for Overcoming the Resistance Mechanisms in Oncogene-Addicted Non-Small Cell Lung Cancer. Int J Mol Sci. 2024;25:11214. 10.3390/ijms252011214 PMC11508910 39456995

[5] Sun J, Corradini S, Azab F, Shokeen M, Muz B, Miari KE, et al. IL-10R inhibition reprograms tumor-associated macrophages and reverses drug resistance in multiple myeloma. Leukemia. 2024;38:2355–65. 39215060 10.1038/s41375-024-02391-8 PMC11518999

[6] Sakamoto KM. Can PROTACs cure Leukemia? Leukemia. 2024;38:2552–3. 39327464 10.1038/s41375-024-02427-z PMC11588658

[7] Welsh SJ, Barwick BG, Meermeier EW, Riggs DL, Shi CX, Zhu YX, et al. Transcriptional Heterogeneity Overcomes Super-Enhancer Disrupting Drug Combinations in Multiple Myeloma. Blood Cancer Discov. 2024;5:34–55. 37767768 10.1158/2643-3230.BCD-23-0062 PMC10772542

[8] Patterson DG, Kania AK, Price MJ, Rose JR, Scharer CD, Boss JM. An IRF4-MYC-mTORC1 Integrated Pathway Controls Cell Growth and the Proliferative Capacity of Activated B Cells during B Cell Differentiation In Vivo. J Immunol. 2021;207:1798–811. 34470852 10.4049/jimmunol.2100440 PMC8455452

[9] Chen Z, Wang M, Wu D, Zhao L, Metwally H, Jiang W, et al. Discovery of CBPD-409 as a Highly Potent, Selective, and Orally Efficacious CBP/p300 PROTAC Degrader for the Treatment of Advanced Prostate Cancer. J Med Chem. 2024;67:5351–72. 38530938 10.1021/acs.jmedchem.3c01789

[10] Fink EC, McConkey M, Adams DN, Haldar SD, Kennedy JA, Guirguis AA, et al. CrbnI391V is sufficient to confer in vivo sensitivity to thalidomide and its derivatives in mice. Blood. 2018;132:1535–44. 30064974 10.1182/blood-2018-05-852798 PMC6172563

[11] Sellar RS, Sperling AS, Słabicki M, Gasser JA, McConkey ME, Donovan KA, et al. Degradation of GSPT1 causes TP53-independent cell death in leukemia while sparing normal hematopoietic stem cells. J Clin Invest. 2022;132:e153514. 35763353 10.1172/JCI153514 PMC9374383

[12] Zhong G, Chang X, Xie W, Zhou X. Targeted protein degradation: advances in drug discovery and clinical practice. Signal Transd Target Ther. 2024;9:308. 10.1038/s41392-024-02004-x PMC11539257 39500878

[13] Lu G, Middleton RE, Sun H, Naniong M, Ott CJ, Mitsiades CS, et al. The myeloma drug lenalidomide promotes the cereblon-dependent destruction of Ikaros proteins. Science. 2014;343:305–9. 24292623 10.1126/science.1244917 PMC4070318

[14] Xiao Z, Song S, Chen D, van Merkerk R, van der Wouden PE, Cool RH, et al. Proteolysis Targeting Chimera (PROTAC) for Macrophage Migration Inhibitory Factor (MIF) Has Anti-Proliferative Activity in Lung Cancer Cells. Angew Chem Int Ed Engl. 2021;60:17514–21. 34018657 10.1002/anie.202101864 PMC8362126

