# Supplementary material for: Targeted degradation of CBP/p300 by CBPD-409 exhibits robust anti-myeloma activity
Source: Leukemia. 2026 Jun 16;40(8):1807–11. doi: 10.1038/s41375-026-03004-2 (PMC13421325; doi:10.1038/s41375-026-03004-2)
Supplement: Supplementary file 1 — Supplementary Fig.1 Western blot quantification of CBPD-409 on oncogenic signaling, proliferation, cell cycle progression, and apoptosis pathways in multiple myeloma cells. [file 41375_2026_3004_MOESM1_ESM.docx]

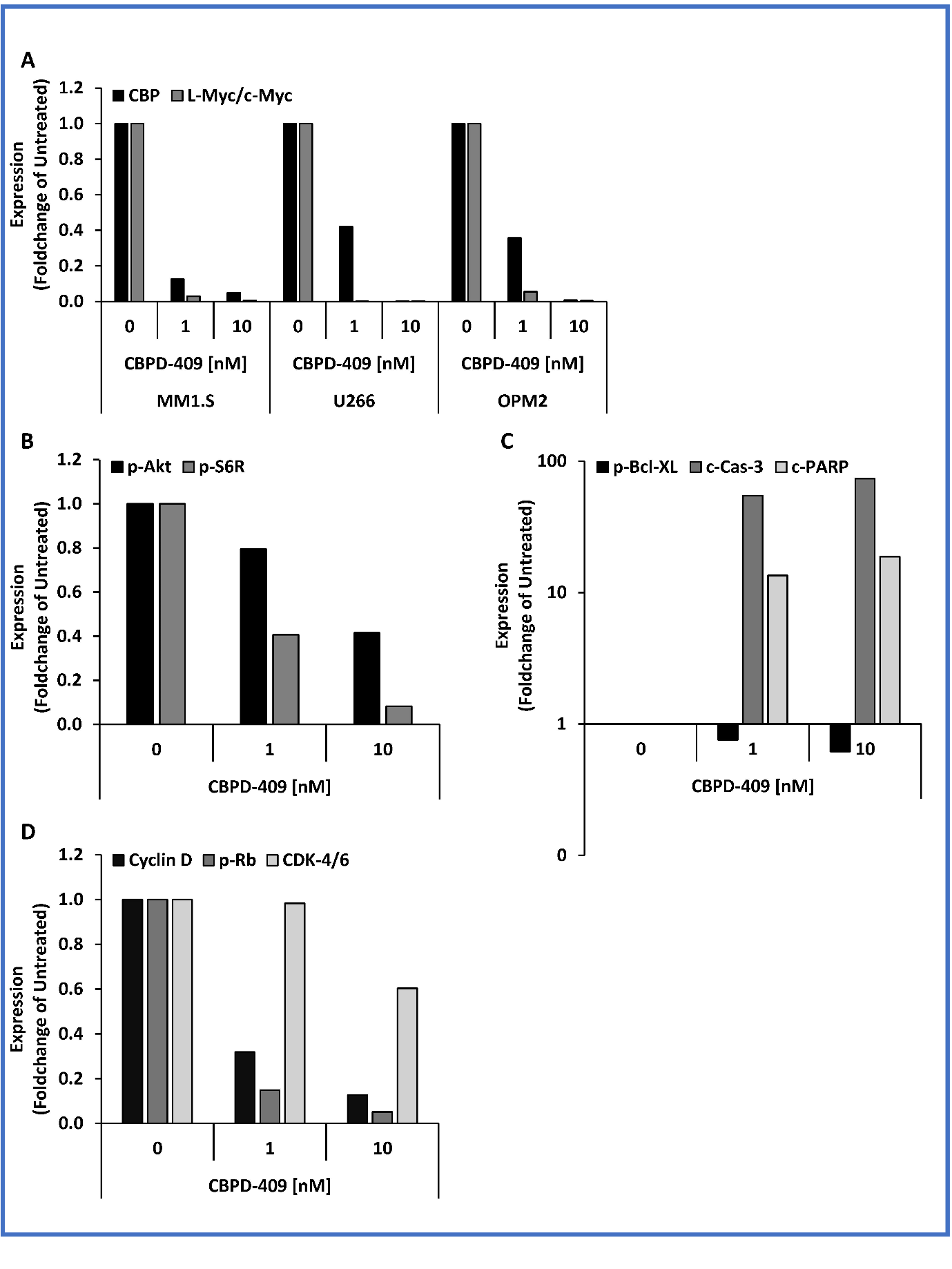


**Supplementary Fig.1 Western blot quantification of CBPD-409 on oncogenic signalling, proliferation, cell cycle progression, and apoptosis pathways in multiple myeloma cells.**

Densitometric quantification of protein expression following treatment with CBPD-409 (1-10 nM) or DMSO for 24 h in multiple myeloma (MM) cell lines (MM.1S, U266, and OPM2). Analysis of signalling proteins including CBP and L-Myc/C-Myc (A); proliferation-associated markers p-AKT and p-S6R (B); cell cycle regulators Cyclin D, p-Rb, and CDK4/6 (C); and apoptosis-related proteins p-Bcl-XL, cleaved caspase-3, and cleaved PARP (D), as determined by immunoblotting of cell lysates. Protein expression levels were normalized to housekeeping proteins.
